# A psychometric and validity study of callous-unemotional traits in 2.5 year old children

**DOI:** 10.1038/s41598-021-87416-3

**Published:** 2021-04-13

**Authors:** Nicola Wright, Andrew Pickles, Helen Sharp, Jonathan Hill

**Affiliations:** 1 Biostatistics Department at the Institute of Psychiatry, King’s College London, 16 De Crespigny Park, Camberwell, London, SE5 8AF UK; 2 Institute of Life and Health Sciences, University of Liverpool, Liverpool, UK; 3 School of Psychology and Clinical Language Sciences, University of Reading, Reading, UK

**Keywords:** Psychology, Human behaviour

## Abstract

Callous-unemotional (CU) traits are associated with severe and stable antisocial behaviour in childhood and adolescence. In order to understand the earliest origins of CU traits we need first to know whether measurement is reliable and valid in young children. This study evaluated the psychometric properties and validity of a CU traits measure generated from existing child problem behaviour scales at age 2.5 years. The participants were members of an epidemiological longitudinal study starting in pregnancy. Items from the Antisocial Process Screening Device and other problem behaviour scales were subjected to exploratory and confirmatory factor analysis. Structural equation modelling was used to test whether age 2.5 CU traits showed incremental validity in predicting aggression at age 5. The CU measure showed acceptable psychometric properties, factorial invariance by sex and good stability. Incremental prediction to later aggression was evident in girls, whereas boys showed strong continuity in aggression not found for girls.

## Introduction

Problems of oppositionality and aggression appearing in early childhood confer a substantially increased risk of later antisocial behaviour disorders and a wide range of psychiatric disorders including depression, anxiety and substance misuse^1^. These early onset ‘life course persistent’ conduct problems share poor long term outcomes and it is likely that there is heterogeneity of risk factors and underlying processes^2^. There is much current interest in a possible subgroup of conduct disordered children who show a lack of concern for the feelings of others and lack of guilt or remorse^3^. In adults these traits are considered part of the affective dimension of psychopathy, and when applied to children they have been labelled as ‘callous-unemotional traits’ (CU traits). The conduct problems in children with CU traits, compared to conduct problems in the absence of CU traits, have been linked to more severe and stable antisocial behaviour in childhood and adolescence^4^ and with more severe violent and aggressive behavior^5^ supporting both the validity of the construct and its importance to understanding persistent and serious antisocial behaviours. Evidence suggests there may be distinct developmental processes contributing to the development of conduct problems with and without CU traits, which has important implications for targeted intervention. For example, conduct problems in children with CU traits, compared to conduct problems in the absence of CU traits, have been found to be more highly heritable^6^ and less influenced by negative parenting practices^7^.

Early research on CU traits focused on samples aged 5–18 years, however, two domains of developmental research suggest that CU traits may be operating and measurable from age 2 years. First, observational studies have identified that empathy-related behaviours and guilt emerge during the first and second years of life^7,8^ which suggests that meaningful variations in CU traits may be measureable as early as age 2 years. Second, trajectories of elevated aggressive behaviour have also been demonstrated to start in the second year of life^9,10^ and if CU traits play a causal role in the development of persistent aggressive behaviour, then CU traits may be measurable and operating at this early age too. Identifying the earliest age at which CU traits can be reliably measured has important implications for research examining the developmental pathways to and from CU traits and for the development of potential preventative intervention before severe antisocial behaviour develops. For example, interventions targeted at promoting parental expressions of warmth have been associated with reductions in CU traits and conduct problems in children aged 3–6 years^11^.

In this study we sought to examine the reliability and validity of the measurement of CU traits at age 2.5 years. We examined internal consistency reliability and four markers of validity. First, we examined whether items with face validity for the same construct appear to form the expected factors as an index of construct validity, second we tested for measurement invariance by sex at ages 2.5 and 5.0 years to confirm the measures generalisability across sexes, third we used factor analysis to examine discriminant validity of CU traits contrasted with child aggression, and fourth we examined whether CU traits at 2.5 years predicted aggression at 5.0 years accounting for all other pathways as a test of incremental validity.

Early research on CU traits in children employed the Antisocial Process Screening Device (APSD^12^) which contains a 6 item CU subscale. Four of these items, which assess lack of concern for others and for one’s own performance, lack of guilt and lack of emotionality have been adopted in the DSM V to create a ‘limited prosocial emotions’ specifier for conduct disorder diagnosis. The APSD has repeatedly shown poor internal reliability, and so was expanded into the 24-item Inventory of Callous-Unemotional traits (ICU^13^).

Some studies have employed these standard measures of CU traits with younger children. The APSD has not been found to shown acceptable internal reliability in samples of 3–5 year olds^14,15^. Dadds et al.^14^ addressed this with a sample of children age 4–8 years by supplementing the APSD with the prosocial items from the Strengths and Difficulties Questionnaire (SDQ). The ICU has acceptable internal reliability at age 3 years but shows a different factor structure to that found with older child and adolescent samples^16,17^. However, both the APSD and ICU have shown validity in predicting future conduct problems and aggression in samples ages 3–5 years^14–17^. The sample used in Kimonis et al.^15^ included a small number of children aged two years, but due to the small size of the sample (N = 49) all children aged 2–5 years were analysed together. To our knowledge no study has examined the APSD or the ICU in a sample of children who were all aged two years. As the APSD and ICU were designed for older children several of the items are developmentally inappropriate for younger children (e.g. “APSD: is good at keeping promises”, “ICU: is concerned about being on time”). Although preschool versions of both scales have been suggested, the only adaption involves replacing reference to “school-work” with “structured activities”, rather than a thorough approach to re-writing the items to be more appropriate for preschool age children. Further, the ICU 12-item solution which has shown the best fit in pre- and early school-age samples^16,18,19^ omits a number of items assessing unconcern about performance, and this component of CU traits seems less relevant to young children. The 12-item solution also omits all but one of the items designed to assess unemotionality. Recently, Colins et al.^20^ developed a scale to assess the full construct of psychopathy in children aged 3–5 years. The Child Problematic Traits Inventory comprises three dimensions assessing callous-unemotional traits, grandiose-deceitfulness and impulsiveness/need for stimulation. The scale shows good internal reliability, and cross-sectional evidence for the validity of the scale and subscales in relation to aggression in cross-section has been provided^21^.

A frequently used method to assess CU traits in young children has been to generate ‘homegrown’ scales using items from existing child problem behaviour measures, this approach benefits from using items designed for use with preschool children and provides the opportunity to analyse CU traits in existing longitudinal datasets. Willoughby and colleagues^22^ developed a five-item CU traits scale using the Child Behaviour Checklist^23^. The authors selected five items from the CBCL which assessed lack of guilt, lack of emotionality (with items specifically assessing lack of affection, unresponsiveness to affection and lack of fear of getting hurt) and unresponsiveness to punishment. They used confirmatory factor analysis to test a three-factor model with the ODD and ADHD subscale items, demonstrating that the CU traits scale was separable from these other child problem behaviour dimensions. This three-factor structure has since been replicated in two further samples of three year olds^24,25^ and a sample of children aged 27 months^26^. The CU items show small to moderate factor loadings and internal reliability is generally low (0.65^25^; 0.55^22^; 0.55^26^). In a large sample of 3 year olds, parent-report on this scale did not correlate with teacher reported CU traits on the ICU^17^, perhaps reflecting the absence of items assessing lack of concern for others and one’s own performance in the CBCL measure. However, prospective evidence for the validity of the CBCL based measure in relation to teacher reported externalising behaviour or aggression at school age has been provided in three independent samples^24–26^. In two of these studies^25,26^ CU traits was modelled as 3-correlated factors which provides evidence for the incremental validity of this CU traits scale after accounting for ODD and ADHD symptoms. Thus whilst this measure shows rather poor psychometric properties at both age 2 and 3 years, similar evidence for validity has been found for both ages.

In a study with a longitudinal sample assessed at age 2, 3 and 4 years^27^, exploratory factor analysis was used on four of the same CBCL items from Willoughby et al.^22^ with the addition of ‘cruel to animals’ and ‘selfish’, and two items from the Eyberg Problem Inventory^28^ assessing sneakiness and lying. This item selection was based on the conceptualisation of CU traits as encompassing behaviours from the interpersonal dimension of psychopathy as well as the affective, and the measure was labelled deceitful-callous behaviour. EFA’s were conducted at each age point and items which showed acceptable factor loadings at every age were retained. The CBCL items assessing lack of affection and unresponsiveness to affection consistently loaded on a separate factor, and CBCL items cruel to animals was also dropped due to low factor loadings. The measure showed acceptable fit in a CFA, although the age 2 factor loadings for the items were below 0.3 for every item except sneakiness. Hyde et al. reported an unsatisfactory Cronbach’s Alpha at age 2 years (α = 0.57 for primary caregiver and α = 0.47 for alternative caregiver report), which was slightly improved by age 3 years (α = 0.64 and α = 0.66) and approaching commonly accepted values for both reporters at age 4 (α = 0.72 and α = 0.66) for their 5 item hybrid measure. The authors concluded that CU traits may not be sufficiently developed to assess at age 2 years. However, this conclusion may be premature given that other studies of 3 and 4 year olds using short measures of CU traits have reported similarly low Cronbach’s Alpha levels. Further, in this study the measure comprised items assessing deceitful or manipulative behaviours which are not typically included in measures of CU traits in childhood, such as the APSD or the ICU affect. Lying and manipulativeness are relevant behaviours seen in adolescence and adulthood but they are likely to require more advanced cognitive abilities than those normally developed at age 2. This developmental and conceptual issue likely contributed to the poorer performance of their measure at age 2 specifically. A key limitation of both the CBCL based and Hyde et al. homegrown CU traits measures is that they do not include items assessing lack of concern for others, which is a core component of CU traits^4,20^.

As previously highlighted, Waller et al.^26^ have provided evidence for the incremental validity of the CBCL CU traits measure at age 2 years, and a number of publications have now produced important findings on the developmental pathways to CU traits using homegrown CU traits measured at age 2 years^29–32^. The poor internal reliability shown in previous studies likely reflects the small number of items used^33^ as well the inclusion of items that may be less relevant to the CU traits construct at this age. In this study, we follow a similar approach to Hyde et al. and use EFA on a pool of items which have face validity for the CU traits construct, focusing on the definition offered by Frick and colleagues which does not include interpersonal features. We follow the approach of Dadds et al. by supplementing a standard measure of CU traits with items from other child problem behaviour scales to improve the internal reliability of the scale. We include all six items from a standard measure of CU traits developed for older children (the APSD) and the CBCL items used by Willoughby et al. and Hyde et al. In addition, items assessing lack of concern for others and lack of prosocial behaviour were selected, from the BITSEA at age 2.5 years, and the SDQ, following Dadds et al.^14^ at age 5 years. We hypothesise that it is possible to generate a CU traits scale at age 2.5 years which shows good psychometric properties, is invariant by sex to allow testing of sex difference hypotheses, and shows incremental validity in relation to physical aggression.

Whether CU traits show sex differences in associations with outcomes has been given relatively little consideration in the literature. It is well-established that boys show higher mean levels of CU traits than girls^17,34,35^. Levels of physical aggression are also lower in girls^36^ even at age 2–3 years^37^. Some studies have examined associations between CU traits and aggression separately for boys and girls and found them to be similar^14,38,39^ although significant sex by CU interactions have been reported by others^34,40^. Few studies have sought to establish that CU measures are invariant across sex before examining associations with outcomes, and therefore any sex difference findings reported may be due to measures operating differently in boys and girls. In this study we test whether the CU traits measure that we generate is invariant across sex, and then examine whether there are sex differences in associations between CU traits and aggression.

In summary, in the current prospective longitudinal study, CU traits and physical aggression were assessed at age 2.5 and again at 5.0 years. The first step was to establish CU traits scales at each age point with satisfactory psychometric properties, including internal reliability and measurement invariance by sex. We examined whether parents could reliably distinguish CU traits and aggression by testing two-factor CU traits and aggression models against one-factor models. CFA was also used to examine whether there were continuities in CU traits from age 2.5 years to 5.0 years. The main analysis used structural equation modelling to examine concurrent and prospective associations between CU traits and physical aggression at 2.5 years and 5.0 years. Our key hypothesis was that age 2.5 CU traits would show incremental validity in predicting physical aggression at age 5.0 years after accounting for age 2.5 physical aggression and all other possible prospective and cross-sectional associations, and that this did not differ by sex of child.

## Method

### Sample

Participants were mothers and children taking part in the Wirral Child Health and Development Study, a prospective epidemiological cohort study starting in pregnancy designed to investigate the earliest origins of childhood conduct problems. The study used a two stage stratified design in which a consecutive general population sample (the ‘extensive’ sample) is used to generate a smaller ‘intensive’ sample stratified by psychosocial risk with more detailed measurement over time and both are followed in tandem^41^. The extensive sample was identified from consecutive first-time mothers who booked for antenatal care at 12 weeks of gestation between 12 February 2007 and 29 October 2008. The booking clinic was administered by the Wirral University Teaching Hospital, which was the sole provider of universal prenatal care on the Wirral Peninsula. The exclusion criteria included age younger than 18 years at the booking appointment, non-English speaking and subsequently infant gross congenital abnormality. Of those approached by study midwives, 68.4% gave consent and completed the measures, yielding an extensive sample of 1233 mothers with surviving singleton babies.

The stratified intensive sample of mothers for more detailed study was generated using mother’s responses to a questionnaire at 20 weeks of pregnancy (recruitment) assessing psychological abuse in their current or recent partner relationship^42^. The stratification variable was chosen for its known association with a variety of risk factors for early child development. The sample stratification design allows the use of weighted analysis to derive estimates of means and coefficients for the whole general population (extensive) cohort from measures available only in the intensive sample. However in this report the weighted least squares mean adjusted (WLSMV) estimator with the stratification variable modelled as an auxiliary variable is used to analyse data from the intensive sample at one time point and the extensive sample at another time point.

The whole cohort of 1233 women of mean age at recruitment of 26.8 years (*SD* = 5.8, range 18–51) and 41.8% of the sample were in the most deprived quintile of UK neighbourhoods^43^. There were 316 mothers recruited to the intensive sample at 32 weeks pregnancy. This report uses questionnaire data collected from the whole cohort who gave data at 20 weeks pregnancy and again at age 5.0 years (*n* = 775, 62.9% of the original cohort), and from the stratified intensive sub-sample at 2.5 years (*n* = 241). Data was also collected from the whole cohort at age 3.5 years, but this report uses data from the more distant age point of 5.0 years to validate the age 2.5 years measure. Nonresponse at age 5 was associated with younger maternal age (*U *(*N* = 1233) = 230,692, *Z* = 8.67, *p* < 0.001) and living in the most deprived quintile of UK neighbourhoods (χ^2^ (1, *N* = 1233) = 19.62, *p* < 0.001). The mean age of the children at the 2.5 year assessment was 30.86 months (*SD* = 2.31, range = 27–42 months) with slightly more girls (*n* = 123) than boys (n = 118), and the mean age of all 775 children whose mothers completed questionnaires at 5.0 years was 58.64 (*SD* = 3.74, range = 49–73 months) with 402 girls and 373 boys. At age 5.0, 80% of mothers were either married or cohabiting, 4.8% had a partner living elsewhere and 15% were single.

### Ethical considerations

Ethical approval for the study was granted by the Cheshire North and West Research Ethics Committee on two occasions for longitudinal data collection, on the 27th June 2006, reference number 05/Q1506/107 for the pregnancy assessment and on 7th June 2010, reference number, 10/H1010/4, for the age 2.5, 3.5 and 5 years assessments. The study has therefore been performed in accordance with the ethical standards laid down in the 1964 Declaration of Helsinki and its later amendments. Written informed consent was obtained from all mothers at recruitment in pregnancy and at every assessment phase in the study. Mothers provided informed consent for their children to take part in the study.

### Measures

#### CU traits

Items were drawn from four different child problem behaviour scales, shown in Table 1. Items were selected based on inclusion in CU traits measures in other studies^13,22,27^ and relevance to the CU traits construct, with a focus on items assessing lack of concern for others, lack of guilt and poverty of affect. All six items from the CU subscale of the APSD were selected for use at both time points (2.5 years and 5.0 years). APSD items are rated on a 3 point scale: 0 = *not true,* 1 = *sometimes true*, 2 = *very true*. Consistent with previous use of the measure with younger samples, the subscale showed somewhat low Cronbach’s Alpha (α = 0.56 at age 2.5 years and α = 0.60 at age 5.0 years). Six items were selected from the CBCL at both time points. The item ‘doesn’t seem to feel guilty after misbehaving’ was not included due to similarity to the APSD item ‘feels bad or guilty when he/she does something wrong’. CBCL items are rated on a 3-point scale: 0 = *not true/never*, 1 = *somewhat true/sometimes*, 2 = *very true/very often*. At age 5.0, all 5 items from the prosocial subscale of the SDQ were selected, based on the University of New South Wales system of combining items from the APSD with the SDQ prosocial items^13^. As the SDQ was not used in this study at age 2.5, one item from the Brief Infant Toddler Socio-emotional Assessment (BITSEA^44^) “tries to help when someone is hurt (for example, gives a toy)” was included at age 2.5 based on its similarity to the SDQ prosocial items. A total of 13 items were selected for age 2.5 years and 17 items for age 5.0 years (shown in Table 1). EFA and CFA were run separately for age 2.5 and 5.0 years and the CU factor composition was allowed to differ at each age to allow for developmental differences in the manifestation of CU traits.

**Table 1:** Items with standardised factor loadings from the two-factor CU traits and aggression models at age 2.5 and 5.0 years and item relevance to the Limited Prosocial Emotions (LPE) specifier.

Items	Age 2.5 Loading	Age 5 Loading	Relevant LPE specifier
**CU traits items**			
APSD 1: Concerned about the feelings of others (R)	0.48	0.47	Callous
APSD 2: Seems motivated to do his/her best in structured activities (R)	0.61		Performance
APSD 3: Is good at keeping promises (R)	0.54	0.47	
APSD 4: Feels bad or guilty when he/she does something wrong (R)	0.43	0.62	Guilt
APSD 5: Keeps the same friends (R)	0.36	0.61	
APSD 6: Does not show emotions			Unemotional
CBCL 14: Cruel to animals	0.99	0.60	
CBCL 58: Punishment doesn’t change his/her behavior	0.62	0.72	
CBCL 67: Seems unresponsive to affection	0.77	0.77	Unemotional
CBCL 69: Selfish or won’t share	0.42		
CBCL 70: Shows little affection toward people	0.48	0.84	Unemotional
CBCL 72: Shows too little fear of getting hurt			Unemotional
BITSEA 22. Tries to help if someone is hurt (R)	0.69		Callous
SDQ 1: Considerate of other people’s feelings (R)		0.75	Callous
SDQ 4: Shares readily with other children (R)		0.53	Callous
SDQ 9: Helpful if someone is hurt, upset or feelings ill (R)		0.57	Callous
SDQ 17: Kind to younger children (R)		0.60	Callous
SDQ 20: Often volunteers to help others (R)		0.46	Callous
**Aggression items**			
Hits other children	0.76	0.87	
Bites other children/Kicks other children	0.75	0.87	
Gets in many fights/Physically attacks others	0.87	0.90	

CBCL = Child Behavior Checklist (CBCL), APSD = Anti-Social Processes Screening Device, BITSEA = Brief Infant Toddler Social and Emotional Assessment (BITSEA), SDQ = Strengths and Difficulties Questionnaire (SDQ), LPE = Limited Prosocial Emotions, Callous = Callous lack of empathy, Guilt = Lack of remorse or guilt, Performance = Unconcern about performance.

#### Physical aggression

Mothers completed a physical aggression questionnaire^30^ at age 2.5 years and 5.0 years. The questionnaire consists of five items previously shown to yield aggression scores with stability from ages 17 to 29 months^37^. The items are shown in Table 1; three out of five items specifically reference aggression towards peers. Each item is rated on a three-point scale: 0 = *not true*, 1 = *somewhat or sometimes true*, 2 = *very true or often true*. The Cronbach’s Alpha in the present sample was adequate for age 2.5 years (α = 0.67) and 5.0 years (α = 0.82).

### Analysis plan

First, the CU items were entered into an exploratory factor analysis for ordinal data (using the weighted least squares mean adjusted estimator [WLSM] and promax rotation) in Mplus version 7^45^ separately for age 2.5 and age 5.0 years. Items with a factor loading > 0.35 were retained. Second, a series of multi-group two-factor CFA, estimated using the WLSMV estimator and Theta parameterization, examined measurement invariance across sex in CU traits and aggression. In model 1 (configural model) the pattern of factor loadings were constrained to be the same for boys and girls, testing that the same items formed the CU and aggression factors across sex. In model 2 (metric model) the individual factor loadings were constrained to be the same, testing whether the contribution of individual items varies by sex (weak factorial invariance). In model 3 (scaler model) the thresholds were also constrained to be the same, to examine whether the items performed the same across sex (strong factorial invariance). Full invariance is demonstrated when the placing of additional constraints on the model does not produce a significant worsening in model fit. The *DIFFTEST* command was used to evaluate whether a substantial change in model fit occurred as a result of imposing additional constraints, as well as the CFI change (ΔCFI). A non-significant chi-square difference test and a small CFI change (in which a decrease is no greater than 0.01) are considered indicative of invariance^46^. If a significant chi-square difference test is found, the modification indices are examined to determine which items failed the strong factorial variance assumption. In the absence of modification indices the individual items are checked for those showing the largest difference between boys and girls. The thresholds of these items are then allowed to vary freely and model fit is re-examined as a test of partial strong factorial invariance.

**Figure 1:**
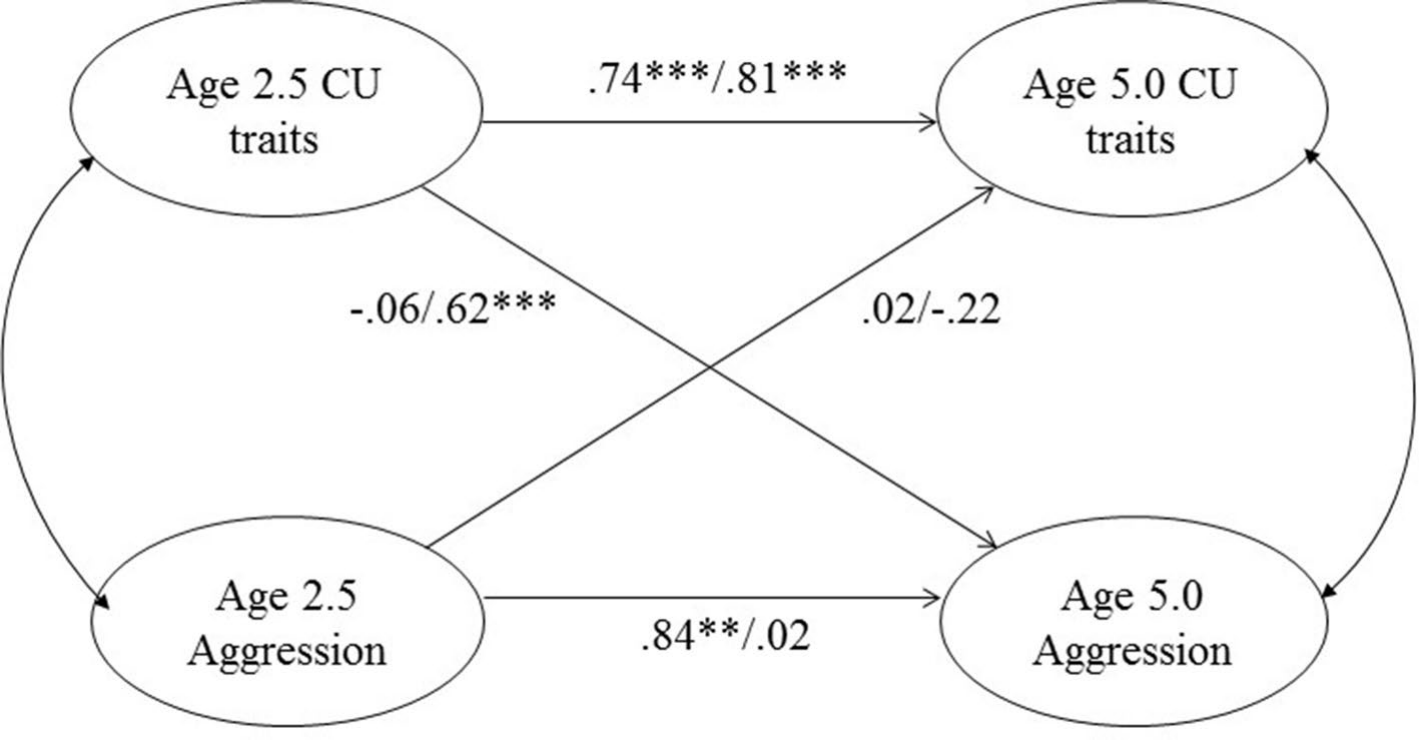
Standardised path estimates for the multivariate model (covariances not shown). Coefficient presented for boys/girls.

Third, two CFA models were estimated and compared using the DIFFTEST command, to test whether a one-factor or two-factor CU and aggression model best fit the data. Finally, two multi-group (by child sex) structural equation models (SEM) for ordinal data were fitted to examine cross-lagged continuity in CU traits and aggression from age 2.5 to 5.0 years, concurrent associations with aggression at each age, and the prospective association between CU traits at age 2.5 and aggression at age 5.0 years, and vice versa. We used the WLMSV estimator in order to include the whole cohort at age 5.0 and the intensive sub-sample at age 2.5 years within a single model. To account for sample stratification of the intensive sub-sample we included the measure on which the stratification was based as an auxiliary variable^47^. We fitted the model of Fig. 1 in which we imposed a common measurement model constraining the factor loadings, measurement errors, and thresholds to be the same across boys and girls and with the factor loading for the first factor fixed at 1 for identification. However, in order to check for sex differences we allowed the means, variances and covariances among the factors to differ by sex of child. The model provided estimates of the effects of time 1 (age 2.5) scores on time 2 (age 5.0) scores, both the simple lagged effects of early CU on later CU traits and aggression on later aggression, and, in order to test the main hypothesis, the cross lagged effects of early CU traits on later aggression, and early aggression on later CU traits. This constrained model gave common estimates of these effects, assuming that a unit change in age 2.5 CU traits or aggression had the same effects on age 5.0 CU traits and aggression in both boys and girls. A second unconstrained model was run allowing the coefficients for girls to be different from those for boys, and the constrained and unconstrained models were then compared using the DIFFTEST.

To avoid numerical problems associated with sparse data in the multivariate model, where endorsement rates were < 1.5%, scores of 1 and 2 were collapsed to create binary variables. This was applied to the CU traits items ‘cruel to animals’ ‘selfish, and ‘shows little affection’ at 2.5 years and 5 years, and to ‘unresponsive to affection’, ‘volunteers to helps others’ (reverse coded), ‘kind to others’ (reverse coded) and ‘considerate to others’ (reverse coded) at 5 years only. Similarly all of the physical aggression items at both ages, except ‘gets in many fights’ at 2.5 years months which had not received a ‘2’ response, were collapsed to create binary variables. Although this generated adequate cell sizes in the sample as a whole, the analytic approach required adequate numbers in both males and females. In females cell sizes were small for ‘gets in many fights’ at age 2.5 years and ‘bites other children’ at age 5 years. “Gets in many fights” was combined with the similar item “physically attacks others”, and “bites other children” was combined with the next rarest item “kicks other children”. The items were combined at both ages to ensure consistency in the physical aggression latent variable across the two ages. Inter-item correlations were added to the CFA and SEM models for items from the same measure to attempt to account for method effects. The item ‘cruel to animals’ showed a negative residual variance at age 2.5 in the multivariate model and so the variance was fixed to 0.01.

The adequacy of all CFA and SEM models was assessed using the Root Mean Square Error (RMSEA) criterion where less than 0.05 is considered a good fit and less than 0.08 considered reasonable fit, and the Comparative Fit Index (CFI) where values above 0.95 indicate good fit and 0.90 reasonable fit^48^.

## Results

### Exploratory factor analysis on the CU items

#### Age 2.5 years

The 13 CU items were entered into an EFA. The APSD item “Does not show feelings or emotions” showed problems with empty cells in the cross-tabulation with two CBCL items (“Shows little affection toward people” and “Seems unresponsive to affection”) and thus was removed from the EFA. Eigenvalues for the first three factors were 4.6, 1.6, and 1.1, and the scree plot supported a one factor solution. The CBCL item “Shows too little fear of getting hurt” gave a factor loading of 0.32 and was dropped, resulting in 11 items at age 2.5 years.

#### Age 5 years

The EFA for the 17 CU items gave eigenvalues of 7.1, 1.6 and 1.2, and the scree plot supported a one-factor solution. The item APSD “Does not show feelings or emotions”, gave a factor loading of 0.26 and so was omitted, resulting in 16 items at age 5.

### Confirmatory factor analyses

#### Age 2.5 years measurement invariance

The 11 items retained at age 2.5 were then tested for measurement invariance across boys and girls using multi-group CFA. Model 1, the configural model, showed good fit (RMSEA = 0.05, CFI = 0.95). The fit improved in model 2 where factor loading invariance was introduced (RMSEA = 0.04, CFI = 0.96) with a non-significant chi-square difference test. Threshold invariance was introduced with model 3 and the fit was largely unchanged (RMSEA = 0.04, CFI = 0.95) the chi-square tests comparing model 3 to model 1 and 2 were both non-significant, demonstrating strong scalar or strong factorial invariance. The full model fit and comparison results are presented in Table S1.

#### Age 5 years measurement invariance

The 16 items retained at age 5 were then tested for measurement invariance across sex. Model 1, the configural model, showed good fit (RMSEA = 0.05, CFI = 0.95). However, the modification indices indicated that items ‘APSD: does his/her best in structured activities’ and ‘CBCL: selfish’ should cross-load on the aggression factor for boys and girls, and ‘CBCL: shows too little fear’ should cross-load for boys. These three items were not considered central to the CU traits construct, and cross-loading with aggression was undesirable given the aim of examining a purely physical aggression outcome, therefore, the items were removed. A further configural model (Model 1b) was tested on the remaining 13 items and showed improved fit (RMSEA = 0.03, CFI = 0.99) with no further modification indices, and so this model was used in further analysis. The introduction of factor loading invariance with model 2 resulted in a further improvement in fit (RMSEA = 0.02, CFI = 0.99) with a non-significant chi-square difference test. However, the introduction of threshold invariance in model 3 resulted in a significant chi-square difference test (*p* = 0.003) and therefore strong factorial invariance was not achieved. There were no modification indices above the minimum value so individual item thresholds were inspected for differences between boys and girls. Items ‘CBCL: hits other children’, ‘CBCL: seems unresponsive to punishment’, ‘APSD: keeps the same friends’ and ‘SDQ: volunteers to help’ all showed a difference of > 0.4, with boys showing a lower threshold than girls on all items apart from ‘volunteers to help’, and so the thresholds for those items were freed. The chi-square test for difference testing between the metric and partial scaler models was now non-significant (*p* = 0.08) therefore we found evidence for partial strong or partial scalar invariance at age 5 years. The full model fit and comparison results are presented in Table S2.

#### One- versus two-factor CU traits and aggression CFA models

We next examined whether mothers could differentiate CU traits and aggression by comparing a one-factor CFA model where all the CU and aggression items loaded on one factor, to the two factor model, using the chi-square DIFFTEST. The model fit statistics and model comparison results are displayed in Table 2. The two-factor model showed the best fit at age 2.5 years (RMSEA = 0.05, CFI = 0.95) and 5.0 years (RMSEA = 0.05, CFI = 0.94) and the chi-square difference tests indicated that the two-factor models showed significantly better fit (*p* < 0.001 for both ages). The standardised factor loadings are displayed in Table 1.

**Table 2:** Fit statistics and results of DIFFTEST for one versus two factor CU and aggression models.

	χ^2^ (df)	CFI	RMSEA	χ^2^ DIFFTEST
**Age 2.5 years**				
1 Factor	104.91 (57)***	0.92	0.06	
2 Factor CU and aggression	83.25 (56)*	0.95	0.05	16.01 (1)***
**Age 5 years**				
1 Factor	677.29 (152)***	0.91	0.07	
2 Factor CU and aggression	275.08 (151)***	0.95	0.05	22.44 (1)***

***p < .001, *p < .05.

### Internal consistency

Cronbach’s Alpha for the CU traits measure at age 2.5 years was α = 0.72 and age 5.0 years α = 0.83. Ordinal Alpha, a more appropriate index of internal consistency for items rated on an ordinal scale, was α = 0.87 for age 2.5 years and α = 0.89 for 5.0 years. Cronbach’s Alpha for the aggression measure after item collapsing was slightly reduced, with α = 0.62 at age 2.5 years and α = 0.82 at age 5 years.

### Sex differences in mean levels

Having established measurement invariance we then used multi-group CFA to test for sex differences in the means on the latent variables. At age 2.5, boys scored significantly higher than girls on CU traits (− 0.34, *p* = 0.041) but not aggression (− 0.32. *p* = 0.198), whereas at age 5 boys scored significantly higher than girls on both CU traits (− 0.32, *p* = 0.002) and aggression (− 0.35, *p* = 0.006).

### Stability for boys and girls

Factor correlations between age 2.5 and 5.0 year CU traits were similar and substantial for boys and girls (0.75, *p* < 0.001 and 0.71 *p* < 0.001) with a somewhat larger association over time for aggression in boys (0.59, *p* < 0.001) than for girls (0.35, *p* < 0.001).

### Multivariate model to examine incremental validity

We fitted the model shown in Fig. 1, and more fully described in the Methods section, to data from both boys and girls, constraining the four path coefficients between the factors to be the same for boys and girls. The model fitted well (RMSEA = 0.03 (CI 0.02–0.03) CFI = 0.93). We next fitted a model in which the path coefficients were allowed to be different, assessing any improvement in fit using the DIFFTEST. While overall fit statistics remained unchanged the DIFFTEST was highly significant, indicating a clear sex difference (*X*^2^ (4) = 10.95, *p* = 0.028) when all the paths were considered. Figure 1 shows the magnitudes of the standardized coefficients where it is evident that the sex differences were of two kinds. There was continuity of aggression from 2.5 to 5.0 years in boys (0.84, *p* = 0.006) but not girls (0.02, *p* = 0.923). By contrast the cross-lagged path from CU traits at 2.5 years to physical aggression at 5.0 years was substantial in girls (0.62, *p* < 0.001) but entirely non-significant for boys (− 0.06, *p* = 0.933). There was no sex difference in continuities from CU traits at 2.5 years to CU traits at 5 years, which was significant for both boys (0.74, *p* < 0.001) and girls (0.81, *p* < 0.001). Cross-sectional correlations between the CU traits and aggression factors at age 2.5 were similar for boys (0.71) and girls (0.58) and this was the case also at age 5 years (0.75 and 0.76 respectively). Combining the various effects together the model estimated that at age 5 years for boys 43% of the variation in the CU factor and 36% of variation in the aggression factor was explained by the measures at age 2.5. The corresponding values for girls were 50% for CU traits and 60% for aggression.

## Discussion

In this study we sought to establish whether CU traits can be measured reliably at 2.5 years, and to assess the validity of measurement at this age as evidenced in an incremental prediction of physical aggression from 2.5 to 5.0 years. We measured CU traits in children aged 2.5 years by supplementing a widely used measure of CU traits, the APSD, with items from other problem behaviour scales for young children. This yielded a CU traits scale invariant across sex with satisfactory psychometric properties that showed strong stability from age 2.5 to 5.0 years for both boys and girls. CU traits and aggression showed significant and substantial associations at age 2.5 and 5.0 years for both boys and girls in line with theory. We found that for girls only, CU traits at 2.5 years predicted physical aggression at 5.0 years after accounting for age 2.5 aggression and all other possible cross-sectional and prospective associations.

As outlined earlier, establishing whether CU traits can be identified in young children is a priority if their role in early onset of aggression is to be studied. There are however substantial issues regarding their measurement that were examined in this study. First, in response to the concern that CU traits may not be identifiable as separable from conduct problems, we showed using CFA that mothers’ ratings of CU traits were separable from ratings of physical aggression at age 2.5 years. This builds on previous findings which have demonstrated that CU traits are distinct from other problem behaviour dimensions at age 27 months and above^13,22,24–26^. Second in order to deal with problems of low internal reliability, we supplemented an established measure of CU traits with items from other child problem behaviour scales and this yielded a measure with satisfactory internal consistency. Third given increasing interest in the possibility that there are sex differences both in the origins and consequences of CU traits, we examined measurement invariance across boys and girls and found strong invariance for the age 2.5 measure and partial strong invariance for the age 5.0 measure. Very few studies have examined measurement invariance by sex for CU traits measures; our findings are consistent with a previous study which demonstrated invariance by sex for a three-correlated factor solution of the ICU at ages 3 and 4 years^17^. Finally we examined incremental validity in the prediction of aggression at 5.0 years from CU traits at 2.5 years, making use of the measurement invariance that we had established to test for sex differences. We found age 2.5 CU traits to predict age 5.0 aggression over and above age 2.5 aggression and all other possible cross-sectional and prospective associations, but in girls only.

The prediction from CU traits at 2.5 years to aggression at 5.0 years additional to the prediction from 2.5 years aggression in girls, but not in boys, needs to be considered in relation to the finding from the same analyses, that the continuity in aggression from 2.5 to 5.0 years was much stronger in boys than in girls. This is consistent with findings from large scale studies of older children that boys are more likely than girls to show stable aggression^10,49^, and with similar evidence from smaller scale studies of preschool children^50,51^. Here we report the same phenomenon in a large preschool sample, and specifically in relation to peer aggression, further supporting the possibility that the emergence and maintenance of early childhood aggression may be underpinned by different processes in boys and girls^52^. Thus one possible interpretation of the failure to show an incremental effect of CU traits in boys is that the extent of change in aggression over the period 2.5 years to 5.0 years, either increasing or decreasing, was so limited that there was little for CU traits to explain. The question is then posed as to whether the key risk processes for aggressive behaviours in boys are to be found before age 2.5 years, with a challenge to identify CU traits or their precursors during infancy or the toddler period. There may also be other explanations for the sex difference such as that the translation of CU traits into aggression is dependent on other influences, for example, deficits in behavioural or physiological inhibitory processes^53,54^, so that even in the absence of main effect, effects may be found in interaction with other variables. Equally the CU traits construct may not be valid in young boys because the relevant empathic processes develop later in boys than girls^55^, or the measure may not be valid because the behaviours that reflect CU traits are not identified in the items of our existing measures. The measure developed in this study comprises only two items assessing unemotionality or shallow/deficient affect (“shows little affection’, “shows too little fear of getting hurt”), both taken from the CBCL and not designed to assess unemotionality as it is conceptualised in adult psychopathy or the DSM with limited prosocial emotions specifier (DSM), which may have impacted the validity of the measure.

Strengths of the study included consecutive recruitment from an antenatal clinic serving a defined geographical area, enabling effect estimates applicable to the general population to be generated. The aggression variable used assessed purely physical aggression, a developmentally appropriate analogous variable to violent behaviour assessed in adolescence. We used item level structural equation modelling (SEM) which first takes account of differing contributions of items, second it enabled all possible prospective pathways to be examined simultaneously, third it allowed measures to operate differently over time or by sex, and fourth it provided tests of sex differences. A limitation of SEM with this sample was that, as a result of some sparse cells, it was necessary to collapse some items to binary variables, and for the aggression latent variable, to combine items. A further limitation of the study was that all of the measures were mother-report questionnaires and so the findings may be influenced by common method variance. The sample is also almost exclusively White British so findings may not generalise to other ethnic groups.

In conclusion, this study showed that a measure of CU traits can be constructed that shows acceptable psychometric properties in children aged 2 years and that measures a construct that is distinct from aggression. Studies of sex differences at this age can be conducted with confidence that items have the same relationship to the latent variable in boys and girls. The marked sex difference, both in stability of aggression and prediction from CU traits, may reflect different pathways to aggression in boys and girls, or differences in validity of the CU traits construct or measure in boys at 2.5 years. Designs of early intervention studies to prevent CU traits will need to account for the possibility that therapeutic approaches may need to vary by sex of the child, and that measurement of early CU traits outcomes may not mean the same in boys and girls.

## Supplementary Information

Supplementary Tables

## Data Availability

Due to ethical constraints supporting data cannot be made openly available. Supporting data are available to bona fide researchers on approval of an application for access. Further information about the data and conditions for access are available at the University of Liverpool Research Data Catalogue: https://doi.org/10.17638/datacat.liverpool.ac.uk/564.

## References

[1] Odgers CL (2008). Female and male antisocial trajectories: From childhood origins to adult outcomes. Dev. Psychopathol..

[2] Hill J (2002). Biological, psychological and social processes in the conduct disorders. J. Child Psychol. Psychiat..

[3] Frick PJ (2009). Extending the construct of psychopathy to youth: Implications for understanding, diagnosing, and treating antisocial children and adolescents. Can. J. Psychiatry.

[4] Frick PJ, Ray JV, Thornton LC, Kahn RE (2014). Can callous-unemotional traits enhance the understanding, diagnosis, and treatment of serious conduct problems in children and adolescents? A comprehensive review. Psychol. Bull..

[5] Kruh IP, Frick PJ, Clements CB (2005). Historical and personality correlates to the violence patterns of juveniles tried as adults. Crim. Justice Behav..

[6] Viding E, Jones AP, Frick PJ, Moffitt TE, Plomin R (2008). Heritability of antisocial behaviour at 9: Do callous-unemotional traits matter?. Dev. Sci..

[7] Kochanska GK, Gross JN, Lin MH, Nichols KE (2002). Guilt in young children: Development, determinants, and relations with a broader system of standards. Child Dev..

[8] Vaish A, Carpenter M, Tomasello M (2009). Sympathy through affective perspective taking and its relation to prosocial behavior in toddlers. Dev. Psychol..

[9] Tremblay RE (2004). Physical aggression during early childhood: Trajectories and predictors. Pediatrics.

[10] Campbell SB (2010). Predictors and sequelae of trajectories of physical aggression in school-age boys and girls. Dev. Psychopathol..

[11] Kimonis ER (2019). Parent-child interaction therapy adapted for preschoolers with callous-unemotional traits: An open trial pilot study. J. Clin. Child Adolesc..

[12] Frick PJ, Hare RD (2001). The Antisocial Process Screening DEVICE (APSD).

[13] Frick, P. J. *Inventory of Callous-Unemotional Traits*. Unpublished rating scale (2004).

[14] Dadds MR, Hawes DJ, Frost A, Fraser J (2005). Disentangling the underlying dimensions of psychopathy and conduct problems in childhood: A community study. J. Consult. Clin. Psychol..

[15] Kimonis ER (2006). Callous-unemotional features, behavioral inhibition, and parenting: Independent predictors of aggression in a high-risk preschool sample. J. Child Fam. Stud..

[16] Kimonis ER (2016). Can callous-unemotional traits be reliably measured in preschoolers?. J. Abnorm. Child Psychol..

[17] Ezpeleta L, Osa NDL, Granero R, Penelo E, Domènech JM (2013). Inventory of callous-unemotional traits in a community sample of preschoolers. J. Clin. Child Adolesc. Psychol..

[18] Hawes SW (2014). Refining the parent-reported inventory of callous-unemotional traits in boys with conduct problems. Psychol. Assess..

[19] Obando, D., Wright, N., & Hill, J. Positive and negative parenting, callous-unemotional traits and oppositional defiant disorder behaviours in preschool Colombian children. Unpublished manuscript.

[20] Colins OF (2014). A new measure to assess psychopathic personality in children: The Child Problematic Traits Inventory. J. Psychopathol. Behav..

[21] López-Romero L, Maneiro L, Colins OF, Andershed H, Romero E (2019). Psychopathic traits in early childhood: Further multi-informant validation of the Child Problematic Traits Inventory (CPTI). J. Psychopathol. Behav.

[22] Willoughby MT, Waschbusch DA, Moore GA, Propper CB (2011). Using the ASEBA to screen for callous unemotional traits in early childhood: Factor structure, temporal stability, and utility. J. Psychopathol. Behav..

[23] Achenbach TM, Rescorla LA (2000). Manual for the ASEBA Preschool Forms & Profiles.

[24] Waller R, Hyde LW, Grabell AS, Alves ML, Olson SL (2015). Differential associations of early callous-unemotional, oppositional, and ADHD behaviors: Multiple domains within early-starting conduct problems?. J. Child Psychol. Psychiatry..

[25] Willoughby MT, Mills-Koonce WR, Gottfredson NC, Wagner NJ (2014). Measuring callous unemotional behaviors in early childhood: Factor structure and the prediction of stable aggression in middle childhood. J. Psychopathol. Behav..

[26] Waller R (2017). Toward an understanding of the role of the environment in the development of early callous behavior. J. Pers..

[27] Hyde LW (2013). Dimensions of callousness in early childhood: Links to problem behavior and family intervention effectiveness. Dev. Psychopathol..

[28] Eyberg SM, Ross AW (1978). Assessment of child behavior problems: The validation of a new inventory. J. Clin. Child Adolesc..

[29] Bedford R, Pickles A, Sharp H, Wright N, Hill J (2015). Reduced face preference in infancy: A developmental precursor to callous-unemotional traits?. Biol. Psychiatry.

[30] Bedford R (2017). The role of infants’ mother-directed gaze, maternal sensitivity, and emotion recognition in childhood callous unemotional behaviours. Eur. Child Adolesc. Psychiatry.

[31] Hyde LW (2016). Heritable and nonheritable pathways to early callous-unemotional behaviors. Am. J. Psychiatry.

[32] Waller R (2016). Heritable temperament pathways to early callous-unemotional behaviour. Br. J. Psychiatry.

[33] Cortina JM (1993). What is coefficient alpha? An examination of theory and applications. J. Appl. Psychol..

[34] Fanti KA, Kimonis ER (2012). Dimensions of juvenile psychopathy distinguish “bullies,” “bully-victims,” and “victims”. Psychol. Violence.

[35] Marsee MA, Silverthorn P, Frick PJ (2005). The association of psychopathic traits with aggression and delinquency in non-referred boys and girls. Behav. Sci. Law.

[36] Card NA, Stucky BD, Sawalani GM, Little TD (2008). Direct and indirect aggression during childhood and adolescence: A meta-analytic review of gender differences, intercorrelations, and relations to maladjustment. Child Dev..

[37] Baillargeon RH (2007). Gender differences in physical aggression: A prospective population-based survey of children before and after 2 years of age. Dev. Psychol..

[38] Essau CA, Sasagawa S, Frick PJ (2006). Callous-unemotional traits in a community sample of adolescents. Assessment.

[39] Silverthorn P, Frick PJ, Reynolds R (2001). Timing of onset and correlates of severe conduct problems in adjudicated girls and boys. J. Psychopathol. Behav..

[40] Thornton LC, Frick PJ, Crapanzano AM, Terranova AM (2012). The incremental utility of callous-unemotional traits and conduct problems in predicting aggression and bullying in a community sample of boys and girls. Psychol. Assess..

[41] Sharp H, Pickles A, Meaney M, Marshall K, Tibu F, Hill J (2012). Frequency of infant stroking reported by mothers moderates the effect of prenatal depression on infant behavioural and physiological outcomes. PLoS ONE.

[42] Moffitt T (1997). Do partners agree about abuse in their relationship? A psychometric evaluation of interpartner agreement. Psychol. Assess..

[43] Noble, M., et al. *The English Indices of Deprivation 2004 (Revised)* (2004).

[44] Briggs-Gowan MJ, Carter AS, Irwin JR, Wachtel K, Cicchetti DV (2004). The Brief Infant-Toddler Social and Emotional Assessment: Screening for social–emotional problems and delays in competence. J. Pediatr. Psychol..

[45] Muthén LK, Muthén BO (2012). Mplus User’s Guide.

[46] Cheung GW, Rensvold RB (2002). Evaluating goodness-of-fit indexes for testing measurement invariance. Struc. Equ. Model..

[47] Graham JW (2003). Adding missing-data-relevant variables to FIML-based structural equation models. Struc. Equ. Model..

[48] Marsh HW, Hau K-T, Wen Z (2004). In search of golden rules: Comment on hypothesis-testing approaches to setting cutoff values for fit indices and dangers to overgeneralizing Hu and Bentler’s (1999) findings. Struct. Equ. Model..

[49] Lee K, Baillargeon RH, Vermunt JK, Wu H, Tremblay RE (2007). Age differences in the prevalence of physical aggression among 5–11-year-old canadian boys and girls. Aggress. Behav..

[50] Alink LRA (2006). The early childhood aggression curve: Development of physical aggression in 10-to 50-month-old children. Child Dev..

[51] Cummings EM, Iannotti RJ, Zahn-waxler C (1989). Aggression between peers in early-childhood—Individual continuity and developmental-change. Child Dev..

[52] Crick NR, Zahn-Waxler C (2003). The development of psychopathology in females and males: Current progress and future challenges. Dev. Psychopathol..

[53] Waller R, Hyde LW, Baskin-Sommers AR, Olson SL (2017). Interactions between callous unemotional behaviors and executive function in early childhood predict later aggression and lower peer-liking in late-childhood. J. Abnorm. Child Psychol..

[54] Wright N, Hill J, Pickles A, Sharp H (2019). Callous-unemotional traits, low cortisol reactivity and physical aggression in children: Findings from the Wirral Child Health and Development Study. Transl. Psychiatry.

[55] Rhee SH (2013). Early concern and disregard for others as predictors of antisocial behavior. J. Child Psychol. Psychiatry.

