# Supplementary material for: A psychometric and validity study of callous-unemotional traits in 2.5 year old children
Source: Sci Rep. 2021 Apr 13;11:8065. doi: 10.1038/s41598-021-87416-3 (PMC8044146; doi:10.1038/s41598-021-87416-3)
Supplement: Supplementary file 1 — Supplementary Tables [file 41598_2021_87416_MOESM1_ESM.docx]

**Supplementary material for “A psychometric and validity study of callous-unemotional traits in 2.5 year old children”**

**Nicola Wright*^1^, Andrew Pickles^1^, Helen Sharp^2^, Jonathan Hill^3^**

^1^Biostatistics Department at the Institute of Psychiatry, King’s College London.

^2^Institute of Life and Health Sciences, University of Liverpool.

^3^School of Psychology and Clinical Language Sciences, University of Reading.

*Corresponding author: Dr Nicola Wright, Biostatistics Department at the Institute of Psychiatry, King’s College London, 16 De Crespigny Park, Camberwell, London, SE5 8AF.

**S1: additional information for the CFA and SEM models**

**Table S1:** Age 2.5 years CFA models testing measurement invariance across sex

|  | **Parameters** | **Chi2(df)** | ***p*** | **RMSEA** | **RMSEA 90% C. I** | **CFI** |
| --- | --- | --- | --- | --- | --- | --- |
| Model 1: configural | 114 | 141.70(110) | .023 | .05 | .02 - .07 | .95 |
| Model 2: metric | 100 | 148.17(124) | .069 | .04 | .00 - .06 | .96 |
| Model 3: scalar | 83 | 167.28(141) | .065 | .04 | .00 - .06 | .95 |
| **Model 1 vs Model 2** |  | 14.89(14) | .444 |  |  |  |
| **Model 1 vs Model 3** |  | 31.88(31) | .422 |  |  |  |
| **Model 2 vs Model 3** |  | 14.76(17) | .612 |  |  |  |

**Table S2:** Age 5.0 years CFA models testing measurement invariance across sex

|  | **Parameters** | **Chi2(df)** | ***p*** | **RMSEA** | **RMSEA 90% C. I** | **CFI** |
| --- | --- | --- | --- | --- | --- | --- |
| Model 1a: configural | 166 | 471.88(232) | .001 | .05 | .05 - .06 | .95 |
| Model 1b: configural **(modified)** | 126 | 199.68(160) | .018 | .03 | .01 - .04 | .99 |
| Model 2:  metric | 113 | 195.97(113) | .111 | .02 | .00 - .03 | .99 |
| Model 3a: scalar | 92 | 237.69(194) | .018 | .02 | .01 - .03 | .99 |
| Model 3b:  Scalar **(modified)** | 190 | 218.99 | .073 | .02 | .00 - 03 | .99 |
| **Model 1b vs Model 2** |  | 9.38(13) | .744 |  |  |  |
| **Model 1b vs Model 3a** |  | 50.22(21) | .003 |  |  |  |
| **Model 1b vs Model 3b** |  | 28.89(3) | .523 |  |  |  |
| **Model 2 vs Model 3b** |  | 25.76(17) | .079 |  |  |  |
